# Sensory immersion vs. cultural reflection: a multi-factor analysis of dual-process mechanisms in digital heritage engagement

**DOI:** 10.3389/fpsyg.2026.1767797

**Published:** 2026-04-10

**Authors:** Cun Shang, Gangqiang Zheng, Ying Xue, Wenxiang Liu

**Affiliations:** 1 School of Landscape Architecture and Art, Xinyang Agriculture and Forestry University, Xinyang, China; 2 College of Art and Design, Wuhan University of Technology, Wuhan, China; 3 College of Art and Design, Yeungnam University, Gyeongsan, Republic of Korea

**Keywords:** cultural identity, dual-mode interface, dual-process theory, functional dissociation, virtual place attachment

## Abstract

**Introduction:**

How do users develop deep psychological bonds with digital heritage environments in the absence of physical embodiment? Prior research has often conflated sensory arousal with cognitive identification. Guided by dual-process theory, we propose that digital heritage appropriation operates through two functionally dissociated pathways: a fast, hedonic route (System 1) driven by sensory stimulation and a slow, reflective route (System 2) driven by meaning-making.

**Methods:**

We employed a multi-factor analytical design integrating unstructured semantic analysis with structured psychological modeling. Study 1 used text mining to map the lexical landscape of user feedback. Study 2 (*N* = 348) tested an extended stimulus-organism-response (S-O-R) model using partial least squares structural equation modeling (PLS-SEM) to examine distinct antecedents of hedonic engagement and cultural identity.

**Results:**

Study 1 identified a dual-demand paradox, with a near-parity lexical ratio (1.1:1) between sensory and reflective orientations. In Study 2, technical affordances (e.g., perceived vividness) strongly predicteda hedonic engagement (*β* = 0.376, *p* < 0.001). Notably, narrative quality showed no significant association with hedonic engagement (*β* = 0.044, *p* = 0.483) but significantly predicted cultural identity (*β* = 0.173, *p* = 0.012).

**Discussion:**

The observed dissociation supports a cognitive resource competition account, suggesting that high-fidelity narratives primarily engage the reflective self rather than the sensory self. In this study, ontological displacement is introduced as an interpretive lens to explain how cultural identity may function as a psychological anchor in contexts where physical spatial coordinates are absent, rather than as a directly operationalized mechanism. Practically, these findings support a dual-mode interface strategy that balances interactive agency with distraction-free narrative absorption to promote psychological sustainability.

## 1 Introduction

Digital technologies can now preserve the *form* of heritage through high-fidelity visual simulation; however, enabling users to appropriate its *meaning* remains cognitively demanding (Bekele et al., 2018). As the experience economy increasingly privileges participation over passive viewing (Pine and Gilmore, 1999), virtual museums often face a “novelty cliff”: traffic initially rises with technological curiosity (Wang et al., 2023) but declines as interface familiarity grows (Mao and Cho, 2024). This pattern points to a fundamental embodiment gap. In physical tourism, multisensory immersion and bodily presence provide somatic cues that support place attachment (Fu and Dong, 2025); by contrast, virtual environments often lack bodily markers that anchor the self to place (Zhang et al., 2020). This raises a central question: how do users develop enduring psychological bonds with digital heritage environments in the absence of physical embodiment?

Existing scholarship offers two partially divergent accounts. Human-computer interaction (HCI) research typically foregrounds a hedonic route, in which sensory affordances (e.g., vividness and interactivity) yield immediate gratification via automatic, high-arousal processing consistent with System 1 (Kahneman, 2011; Deng et al., 2023). By contrast, environmental psychology emphasizes a reflective route, whereby narrative, symbolism, and value transmission support social identity and longer-term loyalty through deliberative processing consistent with System 2 (Zort et al., 2023). Yet how these mechanisms coexist within a single virtual ecosystem remains unclear. A persistent conceptual problem is that studies often treat user engagement (a transient, arousal-related state) and cultural identity (a comparatively stable self-concept) as interchangeable outcomes or as sequential steps (Phinney, 1992). It therefore remains unresolved whether these constructs form a linear progression or instead reflect functionally dissociated psychological processes. Specifically, does narrative quality contribute to momentary excitement, or does it operate primarily to support identity construction?

To resolve this ambiguity, we propose a dual-path dissociation model grounded in an initial qualitative inquiry (Study 1). Rather than assuming a unitary experience, exploratory text mining of user narratives reveals a “dual-demand paradox”: users cluster into two orientations-sensory spectacle versus cultural meaning-with lexical density indicating near parity between the two domains. Building on this evidence, we posit that digital heritage appropriation involves the parallel activation of two orthogonal systems within an extended stimulus-organism-response (S-O-R) framework (Mehrabian and Russell, 1974). Specifically, the model delineates a hedonic interface route (System 1) driven primarily by technical stimuli (e.g., perceived vividness and interactive control) and a distinct identity-construction route (System 2) driven by narrative content quality.

We then test this dissociation in a complementary quantitative study (Study 2). Using a multi-factor analytical framework that integrates unstructured semantic evidence from Study 1 with structured psychological modeling in Study 2, we examine whether narrative quality supports identity construction rather than hedonic engagement-a counterintuitive pattern consistent with cognitive resource competition (Sweller, 1988). Collectively, this framework reinterprets cultural identity not merely as an outcome but as a process that can be understood through the lens of ontological displacement-that is, meaning-based anchoring when physical indexicality is reduced. This interpretive lens is proposed to integrate the pattern of empirical findings rather than to introduce a separately operationalized construct. This perspective suggests that users bond with digital heritage environments not through sensory fidelity alone, but through cognitive alignment with values and meaning (building on Fu and Dong, 2025).

## 2 Literature review and hypothesis development

### 2.1 The stimuli

To address conceptual ambiguities in prior S-O-R research (Mehrabian and Russell, 1974), this study draws a clear boundary between technological inputs (stimuli) and psychological outcomes (organismic states). Rather than treating platform features as fixed technical specifications, we follow Deng et al. (2023) in conceptualizing perceived system affordances as antecedents of psychological presence-external triggers that may selectively activate distinct cognitive pathways. Building on Steuer (1992) and Slater (2003), we operationalize four perceived affordances as stimuli: perceived vividness (PVI), which indexes the “bandwidth” of the sensory channel and the system’s capacity to render high-fidelity environments; perceived interactive control (PIC), which reflects evaluations of mechanisms for manipulating the environment (e.g., zooming and rotation); Interaction Fluency (IF), which captures perceived ease, clarity, and efficiency of navigation (Davis, 1989); and narrative quality (NAQ), which refers to the structural coherence and educational value of storytelling content. Collectively, these affordances are theorized to function as distinct external triggers that may activate parallel-and potentially competing-organismic pathways.

### 2.2 Organism state I

The first organismic state is hedonic interface engagement (HIE). Consistent with the fast pathway in dual-process theory, HIE reflects immediate sensory absorption, positive affect, and low-effort interaction (O’Brien and Toms, 2008). High-fidelity sensory inputs can generate a technological illusion that captures attention via automatic, bottom-up processing (Deng et al., 2023). Accordingly, technical affordances that enhance sensory richness and perceived agency should increase HIE.

*H1*: Perceived vividness (PVI) positively predicts hedonic interface engagement.

*H2*: Perceived interactive control (PIC) positively predicts hedonic interface engagement.

*H3*: Interaction fluency (IF) positively predicts hedonic interface engagement.

Narrative quality and cognitive resource competition. The role of narrative in shaping engagement remains theoretically ambiguous. Narrative Transportation Theory suggests that compelling stories can intensify engagement (Green and Brock, 2000). However, interactive digital heritage experiences impose concurrent demands on interface manipulation (motor control), sensory exploration (visual processing), and content comprehension (semantic processing). Heritage narratives also serve value transmission, which relies more heavily on slow, reflective processing (System 2) (Zort et al., 2023). This combination may elevate intrinsic cognitive load (Sweller, 1988), creating cognitive resource competition in which effortful narrative processing draws on resources required for effortless sensory flow. In line with the broader engagement literature, we specify a positive association, while recognizing that a null effect remains plausible under a cognitive bottleneck.

*H4*: Narrative quality (NAQ) positively predicts hedonic interface engagement.

### 2.3 Organism state II

The second organismic state is cultural identity (CID). Following Fu and Luo (2023), CID is conceptualized as a multidimensional construct comprising cognitive, emotional, and behavioral components.

Ontological displacement and virtual place attachment. In metaverse contexts, the absence of physical embodiment weakens bodily cues that typically support place attachment. Building on Fu and Dong (2025), who identify physical place attachment as a core mediator in industrial heritage settings, this study proposes an alternative anchoring mechanism under conditions of reduced physical indexicality. Specifically, we introduce ontological displacement as an interpretive lens. Conceptually, this implies that when physical somatic cues are absent, human cognition relies on cultural meaning and narrative symbols to actively construct a sense of “being there,” transforming a disembodied virtual space into a psychologically meaningful place. Under conditions of reduced physical indexicality, cultural identity may partially substitute for physical place attachment, linking the self to a virtual place through meaning rather than geographical coordinates. In this sense, identity-based meaning structures may provide a psychological anchor in immersive environments, thereby extending the conceptual argument of Chambel (2016). Because identity construction depends on elaborative, reflective processing, narrative quality is expected to be a primary driver (Zort et al., 2023).

*H5*: Narrative quality (NAQ) positively predicts cultural identity.

Technical affordances are theorized to operate primarily as enablers of reflective processing rather than as direct drivers of meaning. Perceived vividness can increase the tangibility of heritage objects, perceived interactive control can enhance psychological ownership through agentic interaction, and interaction fluency can reduce operational friction. By lowering the cognitive costs of operating the interface, these affordances may free attentional resources for identity-related processing.

*H6*: Perceived vividness (PVI) positively predicts cultural identity.

*H7*: Perceived interactive control (PIC) positively predicts cultural identity.

*H8*: Interaction fluency (IF) positively predicts cultural identity.

### 2.4 Response: satisfaction and continuance intention

At the response level, satisfaction (SAT) is conceptualized as a dual-sourced evaluative state that integrates both hedonic enjoyment (System 1) and eudaimonic identity confirmation (System 2). Accordingly, both HIE and CID are expected to contribute to satisfaction.

*H9*: Hedonic interface engagement (HIE) positively predicts satisfaction.

*H10*: Cultural identity (CID) positively predicts satisfaction.

Consistent with information systems continuance models (Bhattacherjee, 2001) and extending heritage support behavior research (Fu and Dong, 2025), satisfaction is positioned as a proximal driver of continuance intention (CUI). Return intention is expected to increase when sensory pleasure and identity resonance cohere into a satisfying overall experience.

*H11*: Satisfaction (SAT) positively predicts continuance intention (CUI).

*H12*: Satisfaction mediates the effects of both hedonic interface engagement and cultural identity on continuance intention.

## 3 Methodology

### 3.1 Research design and data collection

A sequential mixed-methods design was employed to rigorously test the dual-path functional dissociation model. The quantitative phase (Study 2) was informed by the thematic structure identified in the preliminary unstructured text mining (Study 1), ensuring that the measured constructs possessed high ecological validity.

For the quantitative survey, we operationalized technical stimuli as “antecedents of psychological presence” and recruited users from three major Chinese virtual heritage platforms: the Digital Palace Museum, Digital Dunhuang, and Sanxingdui Digital Museum. These platforms were purposefully selected as they exemplify the distinct classes of stimuli under investigation: they simultaneously offer advanced technological affordances (e.g., high-resolution 360-degree navigability) and rich, curated narrative content (e.g., historical storytelling), making them ideal environments to test the competition between sensory and reflective processing.

An online questionnaire was administered in March 2025. To ensure valid assessment of the high-fidelity immersive affordances (PVI/PIC) central to our hedonic pathway hypothesis, recruitment was purposefully stratified to include a significant proportion of users accessing these platforms via immersive hardware. Consequently, survey links were distributed not only through general social media but specifically within dedicated VR enthusiast communities and the platforms’ immersive experience discussion groups.

To ensure data quality and mitigate the risks of careless responding-a specific concern in unsupervised online surveys-strict quality control measures were implemented. First, responses with completion times falling significantly below the realistic threshold required to read and process the items (“speeders”) were identified and automatically excluded. Second, two embedded attention checks (AC1 and AC2; Oppenheimer et al., 2009) were utilized to identify inattentive respondents; responses failing either check were removed. The final dataset comprised 348 valid responses. As shown in Table 1, the sample skews younger (74.7% aged 25–44) and tech-savvy. Notably, 64.7% of respondents utilized Virtual Reality (VR) headsets, reflecting our targeted sampling strategy aimed at capturing deep immersive experiences rather than mere desktop browsing.

**Table 1:** Demographic characteristics of respondents (*N* = 348).

Variable	Category	*n*	%
Gender	Male	147	42.2
	Female	201	57.8
Age	<18	8	2.3
	19–24	63	18.1
	25–34	173	49.7
	35–44	87	25.0
	45+	17	4.9
Usage frequency	Rarely	56	16.1
	Occasionally	194	55.7
	Frequently	95	27.3
	Daily	3	0.9
Device used	Virtual reality (VR) headset	225	64.7
	Desktop	23	6.6
	Mobile/tablet	100	28.7

The final dataset comprised 348 valid responses. To further assess sample adequacy for the structural model, we conducted a supplementary power-based sample size check using the standard G*Power parameterization commonly reported in PLS-SEM studies. Assuming four primary predictors, a medium effect size (*f*^2^ = 0.15), *α* = 0.05, and statistical power = 0.95, the recommended minimum sample size was 129. Our final sample of 348 substantially exceeded this benchmark, supporting the stability of the PLS-SEM estimations.

### 3.2 Measures and instruments

All constructs were measured using multi-item, five-point Likert scales (1 = strongly disagree; 5 = strongly agree). To ensure construct validity, items were adapted from established frameworks and translated using standard back-translation procedures (Brislin, 1970).

Regarding stimuli (affordances), we measured users’ perceived technological affordances. Perceived Vividness (PVI) and Perceived Interactive Control (PIC) were adapted from Steuer (1992) and Witmer and Singer (1998) to capture the richness of the sensory channel and the extent of user control over the experience environment. Interaction fluency (IF), measured with four items adapted from Davis (1989), was used to capture perceived navigational ease, operational clarity, and interaction smoothness. Narrative Quality (NAQ) comprised three items adapted from Zort et al. (2023), with additional item development informed by Green and Brock (2000) and Sun et al. (2008), specifically assessing the coherence, interpretability, and educational value of the storytelling content.

Regarding organismic states, distinct scales were employed to differentiate the dual pathways. Hedonic interface engagement (HIE) was adapted from O’Brien and Toms (2008) and explicitly operationalized as a “System 1” affective state driven by immediate sensory arousal (e.g., “I felt involved”). In contrast, cultural identity (CID) was measured using items adapted from Fu and Dong (2025) to capture “System 2” eudaimonic depth, focusing on the internalization of cultural values and psychological bonding rather than transient excitement.

Finally, regarding outcomes, satisfaction (SAT) was measured as a cumulative fulfillment response (Oliver, 1997), and continuance intention (CUI) was adapted from Bhattacherjee (2001) to reflect long-term behavioral loyalty. (See Supplementary Appendix A for full measurement items).

### 3.3 Data analysis

Data analysis was conducted in two phases corresponding to the sequential mixed-methods design. First, for the qualitative inquiry (Study 1), data were collected from Chinese platforms to ensure ecological validity. Text mining was performed on the original Chinese corpus using the Jieba library (specifically designed for Chinese semantic segmentation), and extracted themes were translated into English for reporting.

Subsequently, quantitative data (Study 2) were analyzed using Partial Least Squares Structural Equation Modeling (PLS-SEM) in SmartPLS 4. PLS-SEM was selected for its robustness with non-normal data and its suitability for prediction-oriented exploratory models (Hair et al., 2017).

Collinearity and Path Independence Check. Given our central hypothesis regarding the functional dissociation of psychological pathways, ruling out statistical artifacts was paramount. We conducted a rigorous variance inflation factor (VIF) analysis to check for common method bias (CMB) and multicollinearity (Kock, 2015; Podsakoff et al., 2003). All VIF values were found to be below 2.0, well below the conservative threshold of 3.3 (see Table 2). This low collinearity provides robust statistical evidence that Hedonic engagement and cultural identity function as distinct, orthogonal constructs, ensuring that the structural path differences reported later reflect genuine psychological mechanisms rather than measurement overlap. Structural paths and indirect effects were validated via bias-corrected bootstrapping with 5,000 resamples (Preacher and Hayes, 2008).

**Table 2:** Descriptive statistics, correlation matrix, and collinearity diagnostics (VIF).

Construct	Mean	SD	1	2	3	4	5	6	7	8	VIF
1. PVI	4.07	0.86	0.825								1.451
2. PIC	4.11	0.84	0.421	0.782							1.444
3. IF	3.90	0.94	0.418	0.435	0.808						1.340
4. NAQ	3.98	0.97	0.497	0.482	0.353	0.822					1.428
5. HIE	3.87	0.96	0.548	0.507	0.454	0.408	0.825				1.671
6. CID	3.70	1.11	0.480	0.425	0.427	0.427	0.346	0.823			1.442
7. SAT	3.83	1.05	0.366	0.367	0.251	0.314	0.425	0.567	0.850		1.621
8. CUI	4.05	0.95	0.329	0.433	0.205	0.317	0.380	0.331	0.525	0.856	

*N* = 348 for quantitative constructs (Study 2). The qualitative analysis (Study 1) was based on a nested subset of valid text responses (*n* = 118). All correlations are significant at *p* < 0.01. Diagonal values are the square roots of AVE; off-diagonal entries are inter-construct correlations. VIF, variance inflation factor; all values are below 2.0, indicating no multicollinearity concerns.

## 4 Results

### 4.1 Exploratory content analysis (Study 1)

To heuristically ground the theoretical premise of the proposed Dual-Path model prior to confirmatory structural equation modeling, we conducted a preliminary analysis of a subset of open-ended user narratives (*n* = 118) extracted from the survey (i.e., respondents who provided optional text feedback). Using Python and the Jieba library for semantic segmentation, we subjected this corpus to automated content analysis via TF-IDF extraction, after applying cultural-heritage-specific dictionary terms and standard stop-word removal, to identify high-weight latent themes.

**Figure 1:**
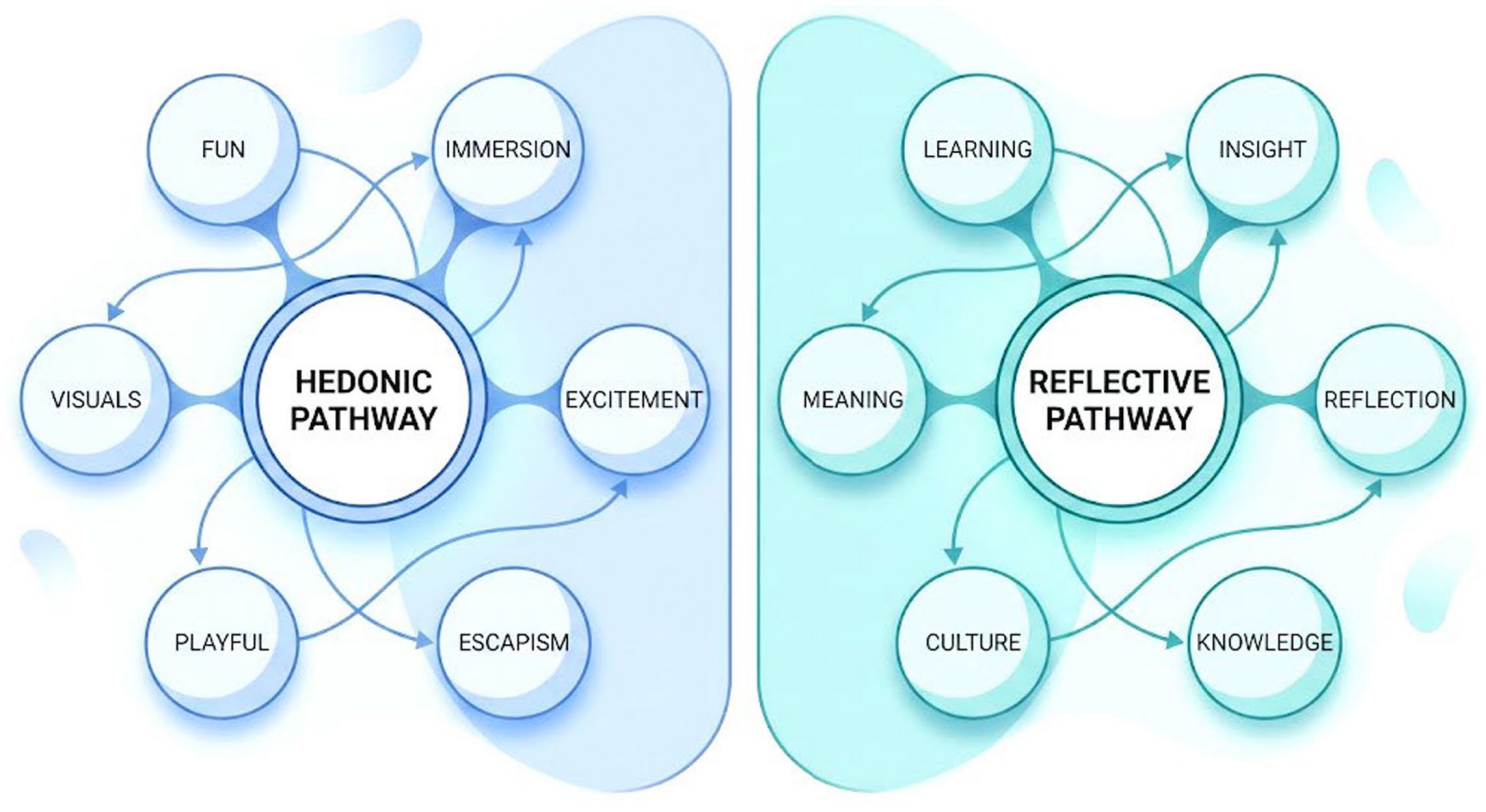
Schematic summary of the semantic bifurcation identified in Study 1.

As visualized in Figure 1, the semantic analysis showed that user feedback was not monolithic but bifurcated into two thematic domains. The hedonic domain (System 1) clustered terms such as “3D visuals,” “immersion,” and “interaction,” reflecting immediate sensory responses. Conversely, the reflective domain (System 2) clustered terms such as “history,” “cultural understanding,” and “national identity.” Importantly, lexical density analysis indicated near parity between these domains, with an approximate token ratio of 1.1:1 between sensory-related and meaning-related terms. This balanced distribution suggests that users treat sensory affordances and cultural cognition as distinct yet equally salient components. This preliminary analysis provided heuristic support for conceptualizing sensory-oriented engagement and meaning-oriented reflection as separable dimensions, thereby motivating the subsequent psychometric validation and structural model testing in Study 2.

This figure provides a conceptual visualization of the two dominant thematic domains identified from the qualitative subset of survey data (*n* = 118) based on TF-IDF-weighted term extraction. The Hedonic Pathway (blue) represents themes related to sensory affordances, whereas the Reflective Pathway (green) represents themes related to cultural cognition.

### 4.2 Measurement model assessment

The measurement model demonstrated adequate reliability and convergent validity across all constructs. As detailed in Table 3, Cronbach’s *α* and composite reliability (CR) values exceeded 0.82, and average variance extracted (AVE) values exceeded 0.50, meeting the criteria recommended by Fornell and Larcker (1981).

**Table 3:** Reliability and convergent validity indicators.

Construct	Items	Cronbach’s *α*	CR	AVE
Perceived vividness	3	0.857	0.865	0.681
Perceived interactive control	3	0.821	0.824	0.611
Interaction fluency	4	0.880	0.882	0.653
Narrative quality	3	0.861	0.862	0.676
Hedonic interface engagement	3	0.867	0.864	0.681
Cultural identity	4	0.891	0.893	0.677
Satisfaction	3	0.881	0.887	0.723
Continuance intention	3	0.890	0.891	0.732

PVI, perceived vividness; PIC, perceived interactive control; IF, interaction fluency; NAQ, narrative quality; HIE, hedonic interface engagement; CID, cultural identity; SAT, satisfaction; CUI, continuance intention.

Collinearity and discriminant validity. Collinearity diagnostics indicated that all variance inflation factor (VIF) values were below 2.0 (max VIF = 1.671), suggesting that multicollinearity is unlikely to bias the estimates (see Table 2). To test whether the hedonic and reflective systems are psychometrically distinct, we examined the heterotrait–monotrait ratio (HTMT). As shown in Table 4, the HTMT ratio between hedonic interface engagement (HIE) and cultural identity (CID) was 0.348. This value is substantially below the conservative 0.85 threshold (Henseler et al., 2015), providing robust evidence that users distinguish between “sensory fun” (System 1) and “cultural meaning” (System 2).

**Table 4:** Discriminant validity (HTMT ratios).

Construct	PVI	PIC	IF	NAQ	HIE	CID	SAT	CUI
PVI								
PIC	0.423							
IF	0.420	0.437						
NAQ	0.499	0.484	0.355					
HIE	0.550	0.509	0.456	0.410				
CID	0.482	0.427	0.429	0.429	0.348			
SAT	0.368	0.369	0.253	0.316	0.427	0.569		
CUI	0.331	0.435	0.207	0.319	0.382	0.333	0.527	

HTMT values below 0.85 indicate adequate discriminant validity (Henseler et al., 2015).

### 4.3 Structural model evaluation

**Figure 2:**
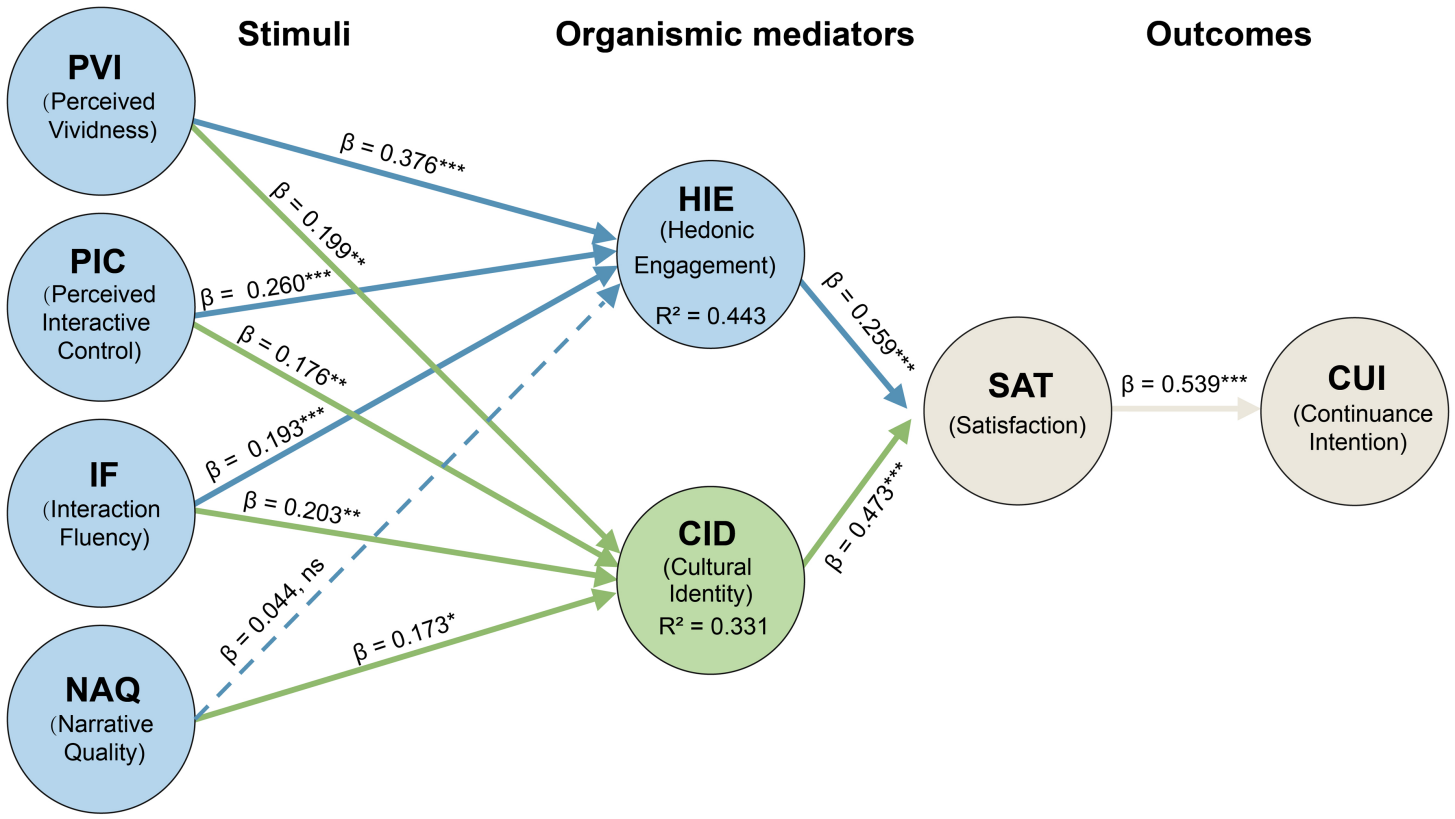
PLS-SEM structural path model results.

Structural model results were interpreted based on path coefficients, effect sizes, and predictive power. As visualized in Figure 2, the model explained 44.3% of the variance in HIE (*R*^2^ = 0.443), 33.1% in CID (*R*^2^ = 0.331), 37.8% in SAT (*R*^2^ = 0.378), and 28.3% in CUI (*R*^2^ = 0.283), indicating substantial predictive power.

The diagram illustrates standardized path coefficients (*β*) and coefficients of determination (*R*^2^). Solid lines denote statistically significant paths, whereas the dashed line indicates the non-significant relationship between narrative quality and Hedonic interface engagement (H4). This pattern is consistent with the proposed dual-path framework and supports the plausibility of a functional dissociation between narrative processing and sensory engagement. **p < 0.001, *p < 0.01, *p* < 0.05; ns = not significant.

*Hedonic pathway* (System 1). Consistent with our affordance-based theorizing, technical stimuli were the dominant predictors of immediate engagement. Perceived vividness (*β* = 0.376, *p* < 0.001), perceived interactive control (*β* = 0.260, *p* < 0.001), and interaction fluency (*β* = 0.193, *p* < 0.001) significantly predicted HIE, supporting H1–H3. Regarding the relative importance of these antecedents, perceived vividness emerged as the strongest technical driver of hedonic interface engagement (*β* = 0.376), followed by perceived interactive control (*β* = 0.260) and interaction fluency (*β* = 0.193).

*Decoupling between sensory immersion and narrative processing* (System 1 vs. System 2). A key finding of this study concerns the relationship between narrative content and immersive engagement in digital heritage environments. Narrative quality did not significantly predict HIE (*β* = 0.044, *p* = 0.483), resulting in the rejection of Hypothesis 4. Importantly, this non-significant path should be interpreted with caution. It does not demonstrate the absence of narrative influence; rather, it is consistent with the proposed dual-process dissociation underlying immersive digital experiences.

Specifically, the observed pattern aligns with a cognitive trade-off perspective in which meaning-oriented narrative comprehension competes for limited attentional resources that would otherwise sustain effortless sensory immersion. Accordingly, in high-fidelity immersive environments, narrative quality may play a stronger role in reflective evaluation and meaning construction (System 2 outcomes) than in immediate sensory-reactive enjoyment (System 1 outcomes).

*Reflective pathway* (System 2). A complementary dissociation pattern emerged for cultural identity. Narrative quality significantly predicted CID (*β* = 0.173, *p* = 0.012), supporting H5. In addition, technical affordances also predicted CID (H5–H7). The significance of interactive control for CID (*β* = 0.176, *p* = 0.009) suggests an embodied agency mechanism: identity resonance strengthens when users actively manipulate heritage objects, fostering psychological ownership rather than passive reception. In relative terms, interaction fluency (*β* = 0.203) and perceived vividness (*β* = 0.199) exhibited slightly stronger technical contributions to cultural identity than perceived interactive control (*β* = 0.176), suggesting that reduced operational friction and perceptual concreteness jointly support reflective identity construction.

#### 4.3.1 Measurement invariance and multi-group robustness check

To assess whether the pooled-sample estimates were potentially confounded by device-related heterogeneity, we conducted an additional measurement invariance assessment and multi-group analysis comparing respondents who used VR headsets (*n* = 225) with those who used non-VR interfaces (desktop/mobile; *n* = 123). Following the MICOM procedure (Henseler et al., 2016), configural invariance was established, as the same indicators, data treatment procedures, and algorithm settings were applied across groups. The permutation results further supported compositional invariance, thereby establishing partial measurement invariance and justifying subsequent comparisons of the structural paths across groups.

A permutation-based PLS-MGA with 5,000 resamples indicated that none of the differences in structural path coefficients between the VR and non-VR groups reached statistical significance (all *p* > 0.05; see Supplementary Table S2). Importantly, the substantive pattern of results remained unchanged across device conditions: Narrative quality did not significantly predict hedonic interface engagement, whereas its association with cultural identity remained positive. Taken together, these findings suggest that the overall dual-path structural pattern is broadly stable across the sampled hardware conditions and that the pooled-sample estimates are unlikely to be materially driven by device-related heterogeneity (Table 5).

**Table 5:** Path coefficients and hypothesis.

Path	*β*	SE	*t*	*p*	Supported
PVI → HIE	0.376	0.084	5.90	<0.001	Yes
PIC → HIE	0.260	0.071	4.08	<0.001	Yes
IF → HIE	0.193	0.063	3.32	<0.001	Yes
NAQ → HIE	0.044	0.067	0.70	0.483	No
PVI → CID	0.199	0.091	2.98	0.003	Yes
PIC → CID	0.176	0.079	2.60	0.009	Yes
IF → CID	0.203	0.071	3.23	0.001	Yes
NAQ → CID	0.173	0.076	2.53	0.012	Yes
HIE → SAT	0.259	0.063	4.63	<0.001	Yes
CID → SAT	0.473	0.066	7.86	<0.001	Yes
SAT → CUI	0.539	0.046	9.22	<0.001	Yes

PVI, perceived vividness; PIC, perceived interactive control; IF, interaction fluency; NAQ, narrative quality; HIE, hedonic interface engagement; CID, cultural identity; SAT, satisfaction; CUI, continuance intention. All *p* < 0.05 are significant. Only H4 (NAQ → HIE) was not supported.

### 4.4 Mediation analysis and behavioral intentions

Bootstrapping (5,000 resamples) was used to test mediation effects. As shown in Table 6, Satisfaction mediated both pathways to continuance intention. The indirect effect of the identity pathway (CID → SAT → CUI = 0.255, 95% CI [0.179, 0.342]) exceeded that of the hedonic pathway (HIE → SAT → CUI = 0.140, 95% CI [0.076, 0.216]). This pattern suggests that sensory engagement may function as an initial “hook,” whereas cultural identity operates as a more durable “anchor” for sustained usage.

**Table 6:** Bootstrapped indirect effects (mediation via satisfaction).

Mediation path	Indirect effect *β*	Boot SE	95% CI lower	95% CI upper	*p*-value	Significant
Hedonic interface engagement → satisfaction → continuance intention	0.140	0.036	0.076	0.216	<0.001	Yes
Cultural identity → satisfaction → continuance intention	0.255	0.041	0.179	0.342	<0.001	Yes

Confidence intervals (CI) not including zero indicate a significant indirect effect at *p* < 0.001.

## 5 Discussion

### 5.1 Theoretical implications

This study supports a dual-path dissociation model and demonstrates functional orthogonality between technology-driven hedonic engagement and culture-driven identity in digital heritage appropriation. Beyond reaffirming the stimulus-organism-response (s-o-r) logic, the multi-factor evidence yields three theoretical advances for understanding how users appropriate heritage in metaverse-like environments.

First, the non-significant association between narrative quality and hedonic engagement (H4) should be interpreted as a substantive psychological boundary condition rather than a methodological artifact. The pattern indicates that users may process cultural narratives primarily via a cognitive-reflective pathway (System 2), rather than via the sensory–reactive pathway (System 1) that underpins immediate enjoyment. This dissociation is consistent with cognitive resource competition: reflective narrative comprehension can draw on cognitive resources that would otherwise sustain effortless sensory flow. In contexts where many users approach digital heritage with a “fast” mindset oriented toward gamified pleasure (Mao and Cho, 2024), imposing high-fidelity narrative demands may increase cognitive load rather than intensify immersion. In other words, interface enjoyment and cultural meaning appear to operate in parallel, rather than unfolding as a single sequential chain.

Second, the significant narrative-to-identity pathway (H5) positions narrative quality as a mechanism of value transmission. Consistent with Zort et al. (2023), storytelling does not merely intensify momentary experience; it supports the internalization of heritage meanings that consolidate into a stable psychological structure → cultural identity → independent of transient arousal. This finding clarifies why narrative quality remains consequential even when it does not increase immediate hedonic engagement: its primary function is reflective self-relevance, not sensory excitation.

Third, building on Fu and Dong (2025), we introduce ontological displacement as an interpretive lens for understanding heritage experience under conditions of reduced physical indexicality. Importantly, ontological displacement is not operationalized or directly measured as a standalone construct; rather, it serves as a theoretical lens that helps integrate the observed pattern of findings. Specifically, the results suggest that meaning-making processes and identity resonance play a central role in sustaining durable digital heritage appropriation.

When bodily co-presence and geographic coordinates are limited, cultural identity may partially substitute for physical place attachment, anchoring the self to a virtual place through meaning rather than location. This implies an ontological shift in sustainable digital appropriation: enduring bonds are less likely to arise from technological spectatorship alone and more likely to emerge through meaning-making processes that affirm the self.

### 5.2 Practical implications

**Figure 3:**
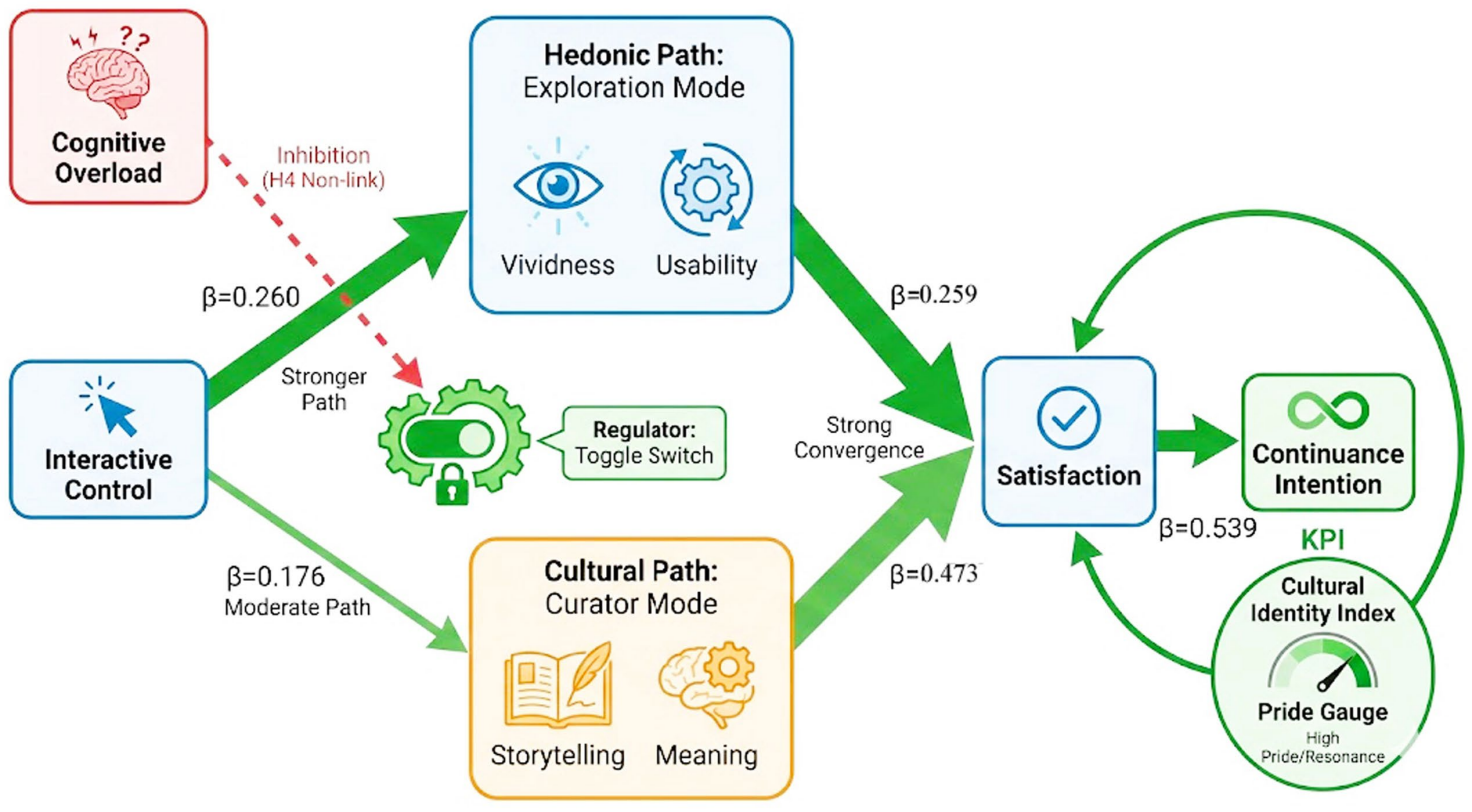
Schematic illustration of the proposed “dual-mode” interface strategy for virtual heritage, derived from the dissociation pattern observed in Study 2 (PLS-SEM).

The distinction between hedonic engagement and cultural identity suggests that virtual heritage design may benefit from more differentiated psychological interventions, rather than relying on a generalized “technology-plus-culture” strategy. On this basis, we propose three practical design implications, as summarized in Figure 3.

First, to address the cognitive tension implied by H4, platforms may consider managing cognitive load through a dual-mode interface structure that separates sensory immersion from narrative interpretation. A default exploration mode (System 1) may emphasize vividness, responsiveness, and fluent navigation in order to support immediate hedonic engagement. In parallel, an optional curator mode (System 2) may serve as a voluntary interpretive layer that reduces sensory distraction and foregrounds narrative comprehension, thereby supporting cultural identity formation. This design logic is broadly consistent with interpretation-oriented or expert-guided presentation modes observed on platforms such as Digital Dunhuang, where historical explanation is given a more central role rather than being overshadowed by excessive interactive stimulation.

Second, perceived interactive control appears to be relevant across both pathways, highlighting the potential importance of embodied agency in digital heritage experiences. Consistent with the embodied cognition perspective on place-related bonding reported by Cai et al. (2025), deeper psychological connection may be more likely when users are able to manipulate digital heritage objects rather than only consume content passively. Accordingly, platforms may consider allocating greater design attention to manipulable artifacts and interactive restoration mechanisms (e.g., virtual disassembly of relics or reconstruction of ruins), rather than relying exclusively on static panoramic presentation. Such features may help cultivate psychological ownership and encourage users to engage with heritage content in a more active and sustained manner.

Third, the long-term development of digital cultural heritage platforms may benefit from evaluation criteria that extend beyond simple visitation counts to include indicators of deeper cultural resonance. The structural model indicates that continuance intention is strongly predicted by satisfaction (SAT → CUI: *β* = 0.539), and that satisfaction is more strongly associated with cultural identity (CID → SAT: *β* = 0.473) than with hedonic engagement (HIE → SAT: *β* = 0.259). Platforms may therefore consider tracking post-experience cultural pride, emotional resonance, and value congruity as complementary outcome indicators. One possible approach would be to develop a Cultural Identity Index as a composite performance indicator incorporating narrative retention, dwell time in interpretation-oriented modules, and post-visit heritage-support intentions. This orientation is consistent with emerging evidence suggesting that sustainable metaverse tourism may depend on moving beyond novelty seeking toward value integration (Jiang et al., 2024; Ray et al., 2025).

More broadly, this shift may also have implications for digital well-being. By fostering deeper cultural resonance, DCH platforms may help reduce the sense of detachment or “rootlessness” that can arise in disembodied virtual environments. In this respect, cultural identity may function as a psychological resource that helps users maintain a more stable sense of connection within digital space.

### 5.3 Limitations and future research

Three limitations warrant attention. First, the sample comprised primarily young, technology-familiar Chinese users; future studies should examine boundary conditions such as cultural traditionality, as stronger traditional values may shape responses to aesthetic and narrative stimuli (Li and Li, 2022). Second, although collinearity diagnostics and discriminant validity were satisfactory, the cross-sectional design limits causal inference; longitudinal studies are needed to track how virtual place attachment develops and stabilizes into sustained loyalty. Third, cognitive resource competition was inferred from self-reports; future work should triangulate this mechanism using neurophysiological measures (e.g., EEG) or behavioral proxies (e.g., dwell-time trade-offs and interaction logs) to quantify resource allocation between narrative processing (System 2) and sensory engagement (System 1) more directly.

## 6 Conclusion

By integrating environmental psychology with human–computer interaction research, this study provides evidence consistent with the dual-path functional dissociation model and offers a more fine-grained explanation of how users psychologically appropriate digital heritage. Moving beyond the technological determinism that characterized earlier studies, our multi-factor analysis suggests that the fast hedonic pathway and the slow reflective pathway operate not merely as isolated features but as distinct psychological mechanisms requiring differentiated design support.

Theoretically, this study challenges the conventional assumption that attachment depends on physical locales. Consistent with the dual-path results, cultural identity may function as a form of meaning-based anchoring that supports virtual place-related bonding when physical cues are limited. We interpret this effect through the lens of ontological displacement rather than as a directly tested mechanism.

Empirically, the observed functional dissociation is consistent with a cognitive resource competition perspective, suggesting that narrative processing may draw on attentional resources that would otherwise sustain sensory immersion. This finding extends existing narrative theory by clarifying that high-fidelity narratives primarily engage the reflective self (System 2), whereas sensory fidelity primarily engages immediate perceptual processing (System 1). This distinction helps explain why richer narrative content does not necessarily yield higher immediate engagement: users may be unable to process both streams simultaneously without some degree of structural decoupling.

From a practical perspective, the findings suggest the value of a dual-mode design strategy that takes users’ cognitive limits into account. The sustainable development of digital heritage platforms may benefit from more targeted interventions, including the use of interaction fluency to engage the fast system during initial engagement and the use of rich, low-distraction narratives to support the slow system in fostering deeper identification. More broadly, user agency appears to serve as a link between these two pathways. For digital heritage to move beyond technological novelty alone, users’ active participation, facilitated by interaction fluency, may function as an important vehicle for meaning construction, encouraging a shift from passive observation toward more active cultural engagement.

## Data Availability

The raw data supporting the conclusions of this article will be made available by the authors, without undue reservation.

## References

[ref1] Bekele M. K. Pierdicca R. Frontoni E. Malinverni E. S. Gain J. (2018). A survey of augmented, virtual, and mixed reality for cultural heritage. J. Comput. Cult. Herit. 11, 1–36. doi: 10.1145/3145534

[ref2] Bhattacherjee A. (2001). Understanding information systems continuance: an expectation-confirmation model. MIS Q. 25, 351–370. doi: 10.2307/3250921

[ref3] Brislin R. W. (1970). Back-translation for cross-cultural research. J. Cross-Cult. Psychol. 1, 185–216. doi: 10.1177/135910457000100301

[ref4] Cai S. Hu Y. He J. Li K. (2025). The impact of embodied cognition on place attachment and supportive behavior toward historic buildings in heritage sites: exploring the moderating role of resident identity climate. Front. Psychol. 16:1702052. doi: 10.3389/fpsyg.2025.1702052, 41307028 PMC12643858

[ref5] Chambel T. (2016). “Interactive and immersive media experiences,” in Proceedings of the ACM International Conference on Interactive Experiences for TV and Online Video, (New York, NY: ACM), 3–10.

[ref6] Davis F. D. (1989). Perceived usefulness, perceived ease of use, and user acceptance of information technology. MIS Q. 13, 319–340. doi: 10.2307/249008

[ref7] Deng Y. Zhang X. Zhang B. Zhang B. Qin J. (2023). From digital museuming to on-site visiting: the mediation of cultural identity and perceived value. Front. Psychol. 14:1111917. doi: 10.3389/fpsyg.2023.1111917, 37034942 PMC10074853

[ref8] Fornell C. Larcker D. F. (1981). Evaluating structural equation models with unobservable variables and measurement error. J. Mark. Res. 18, 39–50. doi: 10.2307/3151312

[ref9] Fu Y. Dong W. (2025). How perceived value, environmental awareness, and social identity shape public support for industrial heritage: the mediating role of place attachment. Front. Psychol. 16:1645646. doi: 10.3389/fpsyg.2025.1645646, 40918296 PMC12412257

[ref001] Fu Y. Luo J. M. (2023). An empirical study on cultural identity measurement and its influence mechanism among heritage tourists. Front. Psychol. 13:1032672. doi: 10.3389/fpsyg.2022.1032672 36743645 PMC9895845

[ref10] Green M. C. Brock T. C. (2000). The role of transportation in the persuasiveness of public narratives. J. Pers. Soc. Psychol. 79, 701–721. doi: 10.1037/0022-3514.79.5.701, 11079236

[ref11] Hair J. F. Hult G. T. M. Ringle C. M. Sarstedt M. (2017). A Primer on Partial least Squares Structural Equation Modeling (PLS-SEM). 2nd Edn. Thousand Oaks, CA: SAGE Publications.

[ref12] Henseler J. Ringle C. M. Sarstedt M. (2015). A new criterion for assessing discriminant validity in variance-based structural equation modeling. J. Acad. Mark. Sci. 43, 115–135. doi: 10.1007/s11747-014-0403-8

[ref13] Henseler J. Ringle C. M. Sarstedt M. (2016). Testing measurement invariance of composites using partial least squares. Int. Mark. Rev. 33, 405–431. doi: 10.1108/IMR-09-2014-0304

[ref14] Jiang S. Zhang Z. Xu H. Pan Y. (2024). What influences users’ continuous behavioral intention in cultural heritage virtual tourism: integrating experience economy theory and S-O-R model. Sustainability 16:10231. doi: 10.3390/su162310231

[ref15] Kahneman D. (2011). Thinking, Fast and Slow. New York, NY: Farrar, Straus and Giroux.

[ref16] Kock N. (2015). Common method bias in PLS-SEM: a full collinearity assessment approach. Int. J. E-Collabor. 11, 1–10. doi: 10.4018/ijec.2015100101

[ref17] Li Y. Li J. (2022). The influence of design aesthetics on consumers' purchase intention: the mediating role of perceived value and the moderating role of traditionality. Front. Psychol. 13:823928. doi: 10.3389/fpsyg.2022.939403 PMC9284263 35846671

[ref18] Mao P. Cho D. M. (2024). Research on an evaluation rubric for promoting user’s continuous usage intention: a case study of serious games for Chinese cultural heritage. Front. Psychol. 15:1300686. doi: 10.3389/fpsyg.2024.1300686, 38425551 PMC10901983

[ref19] Mehrabian A. Russell J. A. (1974). An Approach to Environmental Psychology. Cambridge, MA: MIT Press.

[ref20] O’Brien H. L. Toms E. G. (2008). What is user engagement? A conceptual framework for defining user engagement with technology. J. Am. Soc. Inf. Sci. Technol. 59, 938–955. doi: 10.1002/asi.20801

[ref21] Oliver R. L. (1997). Satisfaction: A Behavioral Perspective on the Consumer. New York, NY: McGraw-Hill.

[ref22] Oppenheimer D. M. Meyvis T. Davidenko N. (2009). Instructional manipulation checks: detecting satisficing to increase statistical power. J. Exp. Soc. Psychol. 45, 867–872. doi: 10.1016/j.jesp.2009.03.009

[ref23] Phinney J. S. (1992). The multigroup ethnic identity measure: a new scale for use with diverse groups. J. Adolesc. Res. 7, 156–176. doi: 10.1177/074355489272003

[ref24] Pine B. J. Gilmore J. H. (1999). The Experience Economy. Boston, MA: Harvard Business School Press.

[ref25] Podsakoff P. M. MacKenzie S. B. Lee J.-Y. Podsakoff N. P. (2003). Common method biases in behavioral research. J. Appl. Psychol. 88, 879–903. doi: 10.1037/0021-9010.88.5.879, 14516251

[ref26] Preacher K. J. Hayes A. F. (2008). Asymptotic and resampling strategies for assessing and comparing indirect effects in multiple mediator models. Behav. Res. Methods 40, 879–891. doi: 10.3758/BRM.40.3.879, 18697684

[ref27] Ray A. Patra S. K. Rastogi A. (2025). Examining the drivers of virtual experiential tourism: from the uses and gratifications theory and stimulus-organism-response theory lens. Asia Pac. J. Tourism Res. 1–28. doi: 10.1080/10941665.2025.2545381

[ref28] Slater M. (2003). A note on presence terminology. Presence Connect 3, 1–5.

[ref29] Steuer J. (1992). Defining virtual reality: dimensions determining telepresence. J. Commun. 42, 73–93. doi: 10.1111/j.1460-2466.1992.tb00812.x

[ref30] Sun P.-C. Tsai R. J. Finger G. Chen Y.-Y. Yeh D. (2008). What drives a successful e-learning? An empirical investigation of the critical factors influencing learner outcomes. Comput. Educ. 50, 1183–1202. doi: 10.1016/j.compedu.2006.11.007

[ref31] Sweller J. (1988). Cognitive load during problem solving: effects on learning. Cogn. Sci. 12, 257–285. doi: 10.1016/0364-0213(88)90023-7

[ref32] Wang M. Liu S. Hu L. Lee J.-Y. (2023). A study of metaverse exhibition sustainability from the perspective of the experience economy. Sustainability 15:9153. doi: 10.3390/su15129153

[ref33] Witmer B. G. Singer M. J. (1998). Measuring presence in virtual environments: a presence questionnaire. Presence 7, 225–240. doi: 10.1162/105474698565686

[ref34] World Medical Association. (2013). Declaration of Helsinki: ethical principles for medical research involving human subjects. JAMA 310, 2191–2194. doi: 10.1001/jama.2013.281053 24141714

[ref35] Zhang G. Chen X. Law R. Zhang M. (2020). Sustainability of heritage tourism: a structural perspective from cultural identity and consumption intention. Sustainability 12:9199. doi: 10.3390/su12219199

[ref36] Zort Ç. Karabacak E. Öznur Ş. Dağlı G. (2023). Sharing of cultural values and heritage through storytelling in the digital age. Front. Psychol. 14:1104121. doi: 10.3389/fpsyg.2023.1104121, 36895745 PMC9990260

