# Supplementary material for: Sensory immersion vs. cultural reflection: a multi-factor analysis of dual-process mechanisms in digital heritage engagement
Source: Front Psychol. 2026 Apr 10;17:1767797. doi: 10.3389/fpsyg.2026.1767797 (PMC13106565; doi:10.3389/fpsyg.2026.1767797)
Supplement: Supplementary file 1 [file Supplementary_File_1.pdf]

Supplementary Material

Appendix A: Survey Measurement Instruments | Appendix B: Data Quality Protocols | Supplementary Table S2: PLS-MGA Results

Appendix A: Survey Measurement Instruments

All constructs were measured using multi-item, five-point Likert scales (1 = Strongly Disagree; 5 = Strongly Agree). Items were adapted from established frameworks and translated using standard back-translation procedures (Brislin, 1970).

Table S1. Construct Items and Sources

Construct	Item No.	Item Content	Literature Source
Perceived Vividness	PVI1	The virtual museum's visual details were so lifelike that I felt as if I was visiting the actual site.	*Witmer & Singer (1998); Steuer (1992)*
	PVI2	The environment was so vivid that my attention was completely captivated by the virtual scene.	
	PVI3	The sensory presentation was realistic enough to make me feel merged with the virtual environment.	
Perceived Interactive Control	PIC1	The virtual museum provides various ways for me to interact with the exhibits.	*Steuer (1992)*
	PIC2	I can flexibly choose the visiting path and operation mode, controlling my experience.	
	PIC3	I can use functions such as zooming and rotating to closely examine exhibit details.	
Interaction Fluency	IF1	The user interface of the virtual museum is intuitive and user-friendly; I found it easy to use.	*Davis (1989)*
	IF2	The interface and operation instructions (such as text or voice guidance) provided by the virtual museum are clear and easy to understand.	
	IF3	I can quickly find exhibits or information that interest me.	
	IF4	The virtual museum runs smoothly and stably on my device (mobile/tablet/VR, etc.).	
Narrative Quality	NAQ1	The stories or background introductions of exhibits in the virtual museum are vivid and interesting.	*Adapted from Zort et al. (2023); items informed by Green & Brock (2000) and Sun et al. (2008)*
	NAQ2	I find the themes and content layout logical and information-rich.	
	NAQ3	The knowledge and educational content obtained were highly fulfilling and met my learning expectations.	
Hedonic Interface Engagement	HIE1	I find using this virtual museum exciting and enjoyable; it feels fun to explore.	*O'Brien & Toms (2008)*
	HIE2	I am highly interested in the interactive activities and features of the virtual museum.	
	HIE3	When using this virtual museum, I feel emotionally involved and entertained.	
Cultural Identity	CID1	I feel a sense of intimacy or curiosity about the cultural heritage displayed in the virtual museum.	*Phinney (1992); Fu & Dong (2025)*
	CID2	During the experience, I gained a deeper understanding of my country's/region's culture.	
	CID3	Through the virtual museum, I feel a stronger sense of cultural identity.	
	CID4	I believe the cultural content presented in the virtual museum is credible and evidence-based.	
Satisfaction	SAT1	Overall, I am satisfied with the virtual museum experience.	*Bhattacherjee (2001); Oliver (1997)*
	SAT2	The immersive presentation and overall environment met my expectations well, leaving me highly pleased.	
	SAT3	Overall, I was very pleased with my experience with this virtual museum.	
Continuance	CUI1	If more relevant content is available in the future, I	*Bhattacherjee (2001)*

Intention		will continue to use the virtual museum.
	CUI2	If new special exhibitions or interactive features are launched, I am very likely to visit again.
	CUI3	I intend to use this virtual museum regularly in the future.

PVI, Perceived Vividness; PIC, Perceived Interactive Control; IF, Interaction Fluency; NAQ, Narrative Quality; HIE, Hedonic Interface Engagement; CID, Cultural Identity; SAT, Satisfaction; CUI, Continuance Intention.

Appendix B: Data Quality Assurance Protocols

To ensure data validity, two screening mechanisms were implemented (N = 348 valid responses; cf. Oppenheimer et al., 2009):

(1) Duration Filtering: Respondents with completion times significantly below the realistic threshold required to read and process the items ("speeders") were identified and automatically excluded.

(2) Attention Checks (AC): Two embedded instructional manipulation checks were included.

AC1: "This is an attention check question. To show you are reading carefully, please select 'Strongly Agree'."

AC2: "For this question, please select 'Neutral' (the third option) to confirm your attention."

Criterion: Failure on either check resulted in case removal.

Supplementary Table S2: Permutation-Based PLS-MGA Results for VR vs. Non-VR Groups

The following table reports the multi-group analysis (PLS-MGA) comparing respondents who used VR headsets (n = 225) with those who used non-VR interfaces -desktop or mobile/tablet (n = 123). Permutation-based PLS-MGA was conducted with 5,000 resamples.

Structural Path	Overall β (N = 348)	VR Group β (n = 225)	Non-VR Group β (n = 123)	Path Diff. (|Δβ|)	p-value (Permutation)	Significance
Hedonic Pathway						
PVI → HIE	0.376***	0.367	0.393	0.026	0.776	ns
PIC → HIE	0.260***	0.262	0.256	0.006	0.958	ns
IF → HIE	0.193***	0.192	0.195	0.003	0.975	ns
NAQ → HIE	0.044 ns	0.099	−0.056	0.155	0.167	ns
Reflective Pathway						
PVI → CID	0.199**	0.256	0.095	0.161	0.071	ns
PIC → CID	0.176**	0.175	0.178	0.003	0.984	ns
IF → CID	0.203**	0.207	0.196	0.011	0.905	ns
NAQ → CID	0.173*	0.197	0.130	0.067	0.508	ns
Response / Outcomes						
HIE → SAT	0.259***	0.262	0.254	0.008	0.962	ns
CID → SAT	0.473***	0.511	0.403	0.108	0.338	ns
SAT → CUI	0.539***	0.506	0.600	0.094	0.397	ns

*Note. Non-VR group combines desktop and mobile/tablet users. p-values were obtained from permutation-based PLS-MGA with 5,000 resamples. None of the between-group differences in structural path coefficients reached statistical significance at the 0.05 level (all $p > 0.05$). ns = not significant. *** $p < 0.001$; ** $p < 0.01$; * $p < 0.05$.*

The substantive pattern of results remained unchanged across device conditions: NAQ did not significantly predict HIE in either group, while its association with CID remained positive. These findings support the overall dual-path structural stability across hardware conditions.
